# Effect of age adjustment on two triage methods

**DOI:** 10.1186/s12873-022-00600-0

**Published:** 2022-03-26

**Authors:** Kirsi Kemp, Janne Alakare, Minna Kätkä, Mitja Lääperi, Lasse Lehtonen, Maaret Castrén

**Affiliations:** 1 Department of Emergency Medicine and Services, Helsinki University Hospital and University of Helsinki Meilahden Tornisairaala, Haartmaninkatu 4, P.O. Box 340, 00029 HUS Helsinki, Finland; 2 Geriatric Acute Care, City of Espoo Karvasmäentie 6, P.O. box, 2704 02070 City of Espoo, Finland; 3 Department of Emergency Medicine, Tampere University Hospital, Ensitie 8, P.O. Box 2000, 33521 Tampere, Finland; 4 Department of Public Health, University of Helsinki and Helsinki University Hospital, Tukholmankatu 2B, P.O.BOX 20, 00014 Helsinki, Finland

**Keywords:** Triage, Older adults, Emergency Severity Index, Emergency Department, Acuity assessment

## Abstract

**Background:**

Most emergency departments rely on acuity assessment, triage, to recognize critically ill patients that need urgent treatment, and to allocate resources according to need. The accuracy of commonly used triage instruments such as the Emergency Severity Index (ESI) is lower for older adults compared to young patients. We aim to examine, whether adjusting the triage category by age leads to improvement in sensitivity without excessive increase in patient numbers in the higher triage categories. The primary outcome measure was 3-day mortality and secondary outcomes were 30-day mortality, hospital admission, and HDU/ICU admissions.

**Methods:**

We gathered data of all adult patients who had an unscheduled visit to any of our three emergency departments within one month. The data was analysed for 3-day mortality, 30-day mortality, hospital admission, and high dependency unit or intensive care unit (HDU/ICU) admission. The analysis was run for both the standard ESI triage method and a local 3-level Helsinki University Hospital (HUH) method. A further analysis was run for both triage methods with age adjustment. Net reclassification improvement values were calculated to demonstrate the effect of age adjustment.

**Results:**

Thirteen thousand seven hundred fifty-nine patients met the study criteria, median age was 57. 3-day mortality AUCs for unadjusted HUH and ESI triage were 0.77 (0.65–0.88) and 0.72 (0.57–0.87); 30-day mortality AUCs were 0.64 (0.59–0.69) and 0.69 (0.64–0.73); hospital admission AUCs were 0.60 (0.68–0.71) and 0.66 (0.65–0.68) and HDU/ICU admission AUCs were 0.67 (0.64–0.70) and 0.82 (0.79–0.86), respectively. Age adjustment improved accuracy for 30-day mortality and hospital admission. With the threshold age of 80, AUCs for 30-day mortality were 0.73 (0.68–0.77) and 0.77 (0.73–0.81) and for hospital admission, 0.66 (0.65–0.67) and 0.72 (0.71–0.73) for the HUH and ESI triage. The effect was similar with all cut off ages.

**Conclusion:**

Moving older adults into a more urgent triage category based on age, improved the triage instruments’ performance slightly in predicting 30-day mortality and hospital admission without excessive increase in patient numbers in the higher triage categories. Age adjustment did not improve HDU/ICU admission or 3-day mortality prediction.

**Supplementary Information:**

The online version contains supplementary material available at 10.1186/s12873-022-00600-0.

## Background

Most emergency departments (ED) rely on acuity assessment, triage, to recognize critically ill patients that need urgent treatment, and to allocate resources according to need. Evidence regarding triage accuracy in older adults is scarce, and the accuracy of triage instruments in comparison to younger adults remains ambiguous.

The emergency severity index (ESI) is a 5-level triage tool, where patients in the most urgent category 1 need immediate life-saving intervention and those in category 5 are estimated not to require any ED resources [1]. A previous study showed that the emergency severity index (ESI) identified less than half of older adults who were in a need of life-saving procedures [2]. Another recent study suggested that age was an independent predictor of ED outcomes, regardless of presenting complaint and ESI triage level [3]. However, two other studies found ESI to be valid for older adults [4, 5], albeit at risk of being undertriaged. One study found the Manchester Triage Scale (MTS) to appear inferior in triaging older adults [6] and another reported increased mortality for older adults independent of triage level with the Rapid Emergency Triage and Treatment System – Adult (RETTS-A) triage [7]. A single study reported validity with the Canadian Triage Acuity Scale (CTAS) for older adults [8]. A recent systematic review listed 18 studies regarding three-level triage systems in adults, none of which reported on older adult triage [9].

In this study, we aim to examine whether adjusting the triage category by age leads to improved sensitivity without excessively increasing patient numbers in the higher triage categories with two separate triage instruments. The primary outcome is 3-day mortality and secondary outcomes are 30-day mortality, hospital admission, and high dependency or intensive care unit (HDU/ICU) admissions.

## Methods

This was a retrospective observational cohort study. We obtained permission for the study from the ethical board of the University of Helsinki (HUS/2678/2017), the Helsinki University Hospital (HUS/280/2019), and Tampere University Hospital (RI8602). We used the STROBE checklist to reduce the risk of bias (Additional file 1: Appendix 1). The data were collected from electronic health care records from Tampere University Hospital (TAYS) and Helsinki University Hospital (HUH). The HUH uses a local 3-level triage method (Additional file 1: Appendix 2), and the TAYS uses the ESI.

### Data collection

We gathered data of all adult patients (18 years and over) who visited three emergency departments between the 1^st^ and the 28^th^ of February 2018. Excluded were all paediatric patients, patients who were dead on arrival, patients who had a scheduled fracture clinic appointment, and patients who were not seen by an ED physician.

For each visit, we recorded the following data: date of birth, gender, time and date of arrival and departure, date of death if within 30 days of the visit, triage category, and hospital and HDU/ICU admissions.

### Analysis

The data were analysed with the IBM Statistical Package for the Social Sciences software (version 25). We used the area under receiving operating characteristic (AUROC) analysis for our outcomes: 3-day mortality, 30-day mortality, hospital admission, and HDU/ICU admission. The outcomes were chosen before running the analysis. The analyses were run for both the standard ESI triage method and a local 3-level HUH method. A second analysis was then run for both triage methods with age adjustment: all patients above a certain cut-off age were moved into a more urgent triage category. The cut-off values used were 65, 70, 75, and 80 years.

Finally, we calculated the net reclassification improvement (NRI) values to demonstrate the effect of applying age adjustment between triage categories [10]. The goal of NRI is to quantify how well the new model reclassifies subjects. Patients who are correctly reclassified are assigned a value of + 1, patients who are incorrectly reclassified are assigned a value of -1, and patients whose classification did not change are assigned a value of 0. The scoring is done separately in both event and non-event groups. The per group NRI is the difference between these values divided by the number of patients; the perfect groupwise NRI would be 1.0 i.e., 100% of the patients in the group were classified better. The overall NRI value is the sum between the groupwise NRIs and can have values between -2 and 2. We used the Bonferroni correction top-values; values below 0.05 were considered significant.

## Results

Within the study period, there were 15 207 recorded visits to our three ED’s. After excluding patients who were dead on arrival (*n* = 36), not seen by an ed physician (*n* = 1797), or who had a scheduled appointment for the outpatient fracture clinic based in the ED (*n* = 438), we had 13374 who met our study criteria. Population characteristics are described in Table 1. A total of 7864 patients were seen at the two Helsinki University Hospital ED’s and 5510 patients at the TAYS ED. The complete list of NRI-values for adjusted triage levels is presented in Additional file 1: Appendix 3.

**Table 1:** Population characteristics

**n**	13374	**Patients triaged by ESI**	5510
**Female**	7119 (53.2%)	1	16 (0.3%)
**Location**	HUH Jorvi ED 3902 (29.2%)	2	541 (9.8%)
	HUH Peijas ED 3962 (29.6%)	3	4041 (73.3%)
	TAYS ED 5510 (41.2%)	4	800 (6.0%)
**30-day mortality**	300 (2.2%)	5	112 (0.8%)
**3-day mortality**	40 (0.3%)		
**Admissions**	4487 (33.6%) ^1^	**Patients triaged by the HUH method**	7864
**HDU/ICU admissions**	675 (5.0%) ^1^	red	122 (1.6%)
**Age**	Mean 56 (SD 22)	yellow	1296 (16.5%)
	Median 57 (IQR 57–74)	green	6446 (82.0%)

### 3-day mortality

**Fig. 1:**
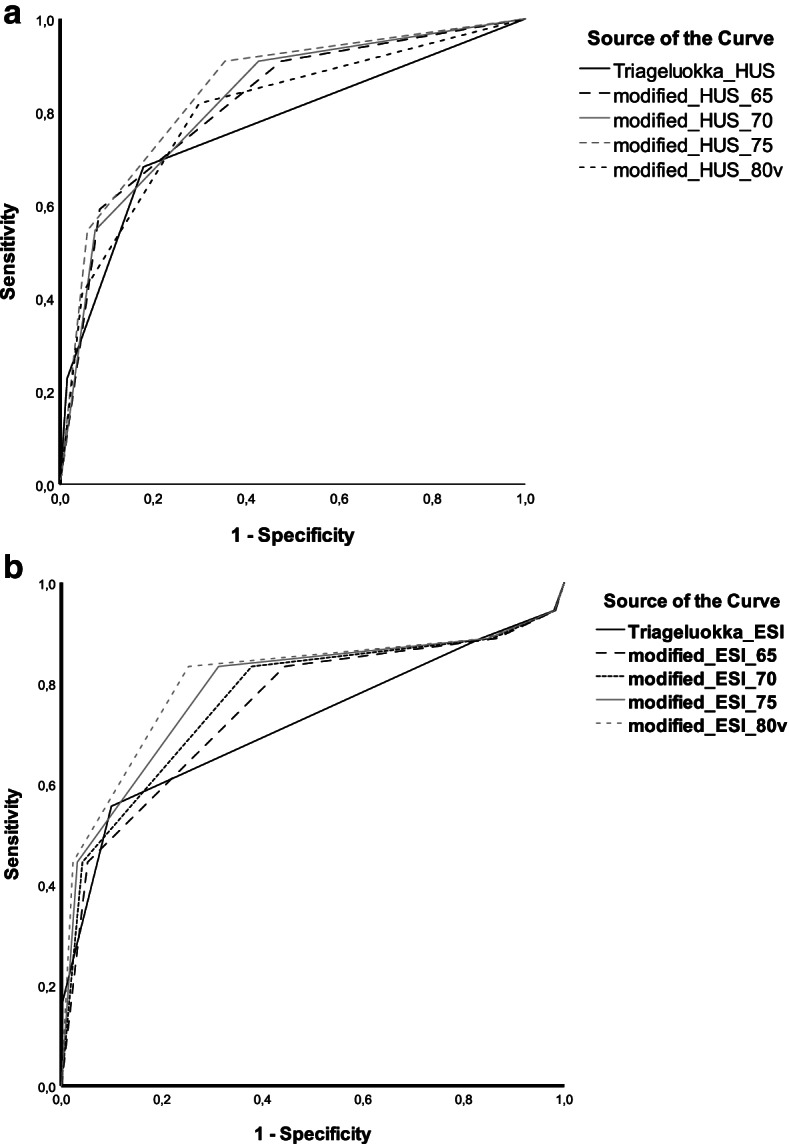
AUC for 3-day mortality prediction for the older adults with HUH triage **a** and ESI **b**. ^a^Six older adults died in the ED and were excluded from admission analysis. HDU/ICU admission data missing for five patients. ^a^ = *p*<0.001

Overall, 3-day mortality was low. AUCs for 3-day mortality were 0.77 (95%CI 0.65–0.88) and 0.72 (95% CI 0.57 – 0.87) for the unadjusted HUH triage and ESI, respectively (Fig. 1). Age adjustment did not improve accuracy for either triage method (Table 2).

**Table 2:** 3-day mortality AUCs for two triage methods with the age adjustment

3-level HUH triage	AUC (95% CI)	ESI triage	AUC (95% CI)
Unadjusted	0.77 (0.65–0.88)	Unadjusted	0.72 (0.57–0.87)
cut off 65 years	0.82 (0.73–0.91)	cut off 65 years	0.75 (0.61–0.89)
cut off 70 years	0.82 (0.74–0.91)	cut off 70 years	0.77 (0.63–0.91)
cut off 75 years	0.85 (0.76–0.93)	cut off 75 years	0.79 (0.65–0.93)
cut off 80 years	0.80 (0.70–0.90)	cut off 80 years	0.81 (0.67–0.94)

With the cut-off of 80 years for ESI, 56% of the patients who died were classified to a higher risk category while 19% of survivors were wrongly classified to a higher category leading to an NRI of 0.37 [95%CI 0.14–0.60, *p* = 0.05]. No other cut-off age led to a significant improvement of the NRI for either triage method (Additional file 1: Appendix 3).

### 30-day mortality

AUCs for 30-day mortality prediction was 0.64 (95%CI 0.59–0.69) and 0.68 (95% CI 0.64–0.73) for the HUH triage and ESI, respectively. Age adjustment improved the performance, there was no significant difference between cut-off ages (Table 3).

**Table 3:** 30-day mortality AUCs for two triage methods adjusted by age

3-level HUH triage	AUC (95% CI)	Emergency Severity Index	AUC (95% CI)
Unadjusted	0.64 (0.56–0.69)	Unadjusted	0.68 (0.64–0.73)
cut off 65 years	0.76 (0.72–0.80)	cut off 65 years	0.77 (0.74–0.81)
cut off 70 years	0.75 (0.71–0.79)	cut off 70 years	0.77 (0.73–0.81)
cut off 75 years	0.76 (0.72–0.80)	cut off 75 years	0.77 (0.73–0.81)
cut off 80 years	0.73 (0.68–0.77)	cut off 80 years	0.77 (0.73–0.81)

With the best cut-off of 75 years for the HUH, 53% of the patients who died were classified to a higher risk category while 21% of survivors were wrongly classified to a higher category leading to an NRI of 0.32 [95%CI 0.24–0.40, *p* < 0.001]. ESI performed similarly. With the 75 years cut-off, 58% of non-survivors were classified better, and 16% of survivors were classified wrongly to a higher category. The NRI was 0.33 [95%CI 0.25–0.41, *p* < 0.001]. All other age adjustments also lead to a statistically significant improvement of both the HUH triage and ESI (Additional file 1: Appendix 3).

### Admission

AUC for hospital admission was 0.60 (95% CI 0.58–0.61) for the unadjusted HUH method and 0.66 (95% CI 0.65–0.68) for unadjusted ESI. Age adjustment improved the performance of both triage methods, there was no significant difference between threshold ages (Table 4).

**Table 4:** Hospital admission AUCs for two triage methods adjusted by age

3-level HUH triage	AUC (95% CI)	ESI triage	AUC (95% CI)
Unadjusted	0.60 (0.58–0.61)	Unadjusted	0.66 (0.65–0.68)
cut off 65 years	0.69 (0.68–0.71)	cut off 65 years	0.74 (0.73–0.76)
cut off 70 years	0.69 (0.68–0.70)	cut off 70 years	0.73 (0.72–0.75)
cut off 75 years	0.68 (0.66–0.70)	cut off 75 years	0.73 (0.71–0.74)
cut off 80 years	0.66 (0.65–0.67)	cut off 80 years	0.72 (0.71–0.73)

With the best cut-off of 65 years for the HUH, 59% of admitted patients were classified to a higher risk category while 21% of discharged patients were wrongly classified to a higher category leading to an NRI of 0.27 [95%CI 0.25–0.29, *p* < 0.001].

ESI performed similarly. With the cut-off at 65 years, 62% of admitted patients were classified better, and 30% of discharged patients were classified wrongly to a higher category. The NRI was 0.32 [95%CI 0.29–0.34, *p* < 0.001]. All other age adjustments also lead to a smaller, but significant improvement of both the HUH triage and ESI (Additional file 1: Appendix 3).

### HDU/ICU admission

AUCs for unadjusted HUH and ESI were 0.67 (95% CI 0.64–0.70) and 0.82 (95% CI 0.79–0.86), respectively. Age adjustment did not improve the performance for either method (Table 5; Additional file 1: Appendix 3).

**Table 5:** HDU/ICU admission AUCS for two triage methods, with the age adjustment

3-level HUH triage	AUC (95% CI)	ESI triage	AUC (95% CI)
Unadjusted	0.67 (0.64–0.70)	Unadjusted	0.82 (0.79–0.86)
cut off 65 years	0.70 (0.67–0.72)	cut off 65 years	0.78 (0.74–0.81)
cut off 70 years	0.69 (0.66–0.72)	cut off 70 years	0.76 (0.73–0.80)
cut off 75 years	0.68 (0.65–0.71)	cut off 75 years	0.76 (0.73–0.80)
cut off 80 years	0.68 (0.65–0.71)	cut off 80 years	0.78 (0.74–0.81)

### Strengths and limitations

Our study included patients from several EDs, and the number of included patients was relatively large, which gives some weight to our findings. A large portion of our patients was triaged by an informal 3-level triage method, which limits the applicability of the results. The triage methods were analysed separately and the improvement in accuracy was at least as good in ESI than in the 3-level system.

Previous studies regarding triage for older adults have reported variable measures from ED mortality to one-year mortality. Studies concerning the general adult population, with a larger number of participants, have commonly reported ED- or in-hospital mortality [9]. As the 3-day mortality in our study was low, a longer study period might be required in future studies to gain stronger data regarding short-term (ED, in-hospital, or 3-day) mortality.

Limitations of the study included retrospective data collection. Some bias related to seasonal variations is possible due to the limited study period. However, a fixed continuous time period was chosen to limit the risk of selection bias. We suggest that any further studies on the topic could address the impact of seasonal variation. Regarding our data, we have had to rely on data previously collected by other staff, occasionally leading to missing data. However, the data available from the EHR’s are reliable and conclusive. Finally, we applied the STROBE checklist to our study to reduce the risk of bias.

## Discussion

According to our results, adjusting triage categories by age did not improve 3-day mortality prediction. Age adjustment improved accuracy for 30-day mortality and hospital admission, which were the outcomes where the original methods were weakest. These findings reflect the results of Ginsburg et al. [3], who found that age was an independent predictor for these outcomes. Our results showed a slight decrease in HDU/ICU admission prediction with age adjustment, however, accuracy remained adequate.

The improvements were similar within the cut-off age intervals. The outcomes for which the age adjustment improved both triage tools i.e., hospital admission and 30-day mortality, were similarly improved by each cut-off age. A previous study suggested that the accuracy of an early warning score improved when combined with age for patients over 80 years. According to our results, a threshold age of 80 was equal to the other tested age limits, and since choosing a high cut-off age would mean a smaller rate of over triage, we suggest that further studies would consider the same age threshold.

Implementing an age-adjusted triage tool in practice would be fairly straightforward. The triage process could be run as-is, and once completed, the triage nurse would check the patient’s age and increase the urgency by one, if over the threshold age. In practice, implementing an age-adjusted triage scale would mean an increased number of older adults in the more urgent triage categories. While this would be an improvement for the older ED patients, it might increase waiting times for patients under the threshold age. Younger patients in the more urgent triage categories would remain urgent, however, and undertriage in the non-elderly patients is less common. Non-elderly patients are also less prone to negative outcomes associated with longer waiting times. Thus, we argue that the negative effect on the younger ED patients would be smaller than the positive effect on the older adults. A prospective implementation study would answer how age adjustment would change the ED flow in general and for each age group.

## Conclusion

Moving older adults into a more urgent triage category based on age, improved the triage instruments’ performance slightly in predicting 30-day mortality and hospital admission without excessive increase in patient numbers in the higher triage categories. Age adjustment did not improve HDU/ICU admission prediction or 3-day mortality prediction. The optimal age threshold remains unclear.

## Supplementary Information

**Additional file 1:** **Appendix 1:** STROBE checklist. **Appendix 2:** Our 3-level triage tool (abbreviated and translated from original Finnish version). **Appendix 3:** NRI values for retriaged patients.

## Data Availability

The datasets are not publicly available due to national juridical restrictions protecting pseudonymized research data. Pseudonymized data is not allowed in the study permission from the ethical board. Further description or analysis of data are available from the authors upon reasonable request.

## Abbreviations

AUROC: Area under the receiver operating characteristics
CTAS: Canadian Triage Acuity Scale
ED: Emergency department
ESI: Emergency severity index
HDU: High dependency unit
HUH: Helsinki University Hospital
ICU: Intensive care unit
MTS: Manchester Triage Scale
RETTS-A: Rapid Emergency Triage and Treatment System – Adult
ROC: Receiver operating characteristics
STROBE: Strengthening the reporting of observational studies in epidemiology
TAYS: Tampere University Hospital

## References

[1] Gilboy N, Tanabe P, Travers D, Rosenau A. Implementation Handbook. Emergency Severity Index version 4. Schaumburg: Emergency Nurses Association; 2020.

[2] Platts-Mills TF, Travers D, Biese K, McCall B, Kizer S, LaMantia M (2010). Accuracy of the Emergency Severity Index Triage Instrument for Identifying Elder Emergency Department Patients Receiving an Immediate Life-saving Intervention. Acad Emerg Med.

[3] Ginsburg AD, Oliveira L, Silva J, Mullan A, Mhayamaguru KM, Bower S (2021). Should age be incorporated into the adult triage algorithm in the emergency department?. Am J Emerg Med.

[4] Baumann MR, Strout TD (2007). Triage of geriatric patients in the emergency department: Validity and survival with the Emergency Severity Index. Ann Emerg Med.

[5] Grossmann FF, Zumbrunn T, Frauchiger A, Delport K, Bingisser R, Nickel CH (2012). At Risk of Undertriage? Testing the Performance and Accuracy of theEmergency Severity Index in Older Emergency Department Patients. Ann Emerg Med.

[6] Brouns SHA, Mignot-Evers L, Derkx F, Lambooij SL, Dieleman JP, Haak HR (2019). Performance of the Manchester triage system in older emergency department patients: a retrospective cohort study. BMC Emerg Med.

[7] Ruge T, Malmer G, Wachtler C, Ekelund U, Westerlund E, Svensson P (2019). Age is associated with increased mortality in the RETTS-A triage scale. BMC Geriat..

[8] Lee JMY, Oh SH, Peck EH, Lee JMY, Park KN, Kim SH (2011). The validity of the Canadian Triage and Acuity Scale in predicting resource utilization and the need for immediate life-saving interventions in elderly emergency department patients. Scandinavian Journal of Trauma, Resuscitation and Emergency Medicine.

[9] Zachariasse JM, van der Hagen V, Seiger N, Mackway-Jones K, van Veen M, Moll HA. Performance of triage systems in emergency care: a systematic review and meta-analysis. BMJ Open. 2019;9(5). 10.1136/bmjopen-2018-026471 PMC6549628 31142524

[10] Pencina M, D’Agostino RBJs, D’Agostino RBJ, Ramachandran V (2008). Evaluating the added predictive ability of a new marker: From area under the ROC curve to reclassification and beyond. Stat med.

