# Supplementary material for: Effect of age adjustment on two triage methods
Source: BMC Emerg Med. 2022 Mar 26;22:52. doi: 10.1186/s12873-022-00600-0 (PMC8961917; doi:10.1186/s12873-022-00600-0)
Supplement: Supplementary file 1 — Additional file 1: Appendix 1: STROBE checklist. Appendix 2: Our 3-level triage tool (abbreviated and translated from original Finnish version). Appendix 3: NRI values for retriaged patients. [file 12873_2022_600_MOESM1_ESM.docx]

# Appendices

## Appendix 1: STROBE checklist

STROBE Statement—checklist of items that should be included in reports of observational studies

|  | Item No. | | Recommendation | Relevant text from manuscript | | | | | |
| --- | --- | --- | --- | --- | --- | --- | --- | --- | --- |
| **Title and abstract** | | 1 | (*a*) Indicate the study’s design with a commonly used term in the title or the abstract | “This was a retrospective observational cohort study” | | | | | |
|  |  |  | (*b*) Provide in the abstract an informative and balanced summary of what was done and what was found | See Abstract | | | | | |
| Background/rationale | | 2 | Explain the scientific background and rationale for the investigation being reported | See background | | | | | |
| Objectives | | 3 | State specific objectives, including any prespecified hypotheses | In this cohort study, aim to show that adjusting triage category for the older adults leads to greater sensitivity without excessive increase patient numbers in the higher triage categories. | | | | | |
| Study design | | 4 | Present key elements of study design early in the paper | “This was a retrospective observational cohort study.” | | | | | |
| Setting | | 5 | Describe the setting, locations, and relevant dates, including periods of recruitment, exposure, follow-up, and data collection | See methods | | | | | |
| Participants | | 6 | (*a*) *Cohort study*—Give the eligibility criteria, and the sources and methods of selection of participants. Describe methods of follow-up | We gathered data of all adult patients who visited three emergency departments between the 1^st^ and the 28^th^ of February 2018. Excluded were all paediatric patients, patients who were dead on arrival, patients who had a scheduled fracture clinic appointment and patients who not seen by and ED physician. | | | | | |
| Variables | | 7 | Clearly define all outcomes, exposures, predictors, potential confounders, and effect modifiers. Give diagnostic criteria, if applicable | The primary outcome is 3-day mortality and secondary outcomes are 30-day mortality, hospital admission and HDU/ICU admissions. | | | | | |
| Data sources/ measurement | | 8* | For each variable of interest, give sources of data and details of methods of assessment (measurement). Describe comparability of assessment methods if there is more than one group | See methods | | | | | |
| Bias | | 9 | Describe any efforts to address potential sources of bias | Our study included patients from several EDs, and the number of included patients was relatively large.  Some bias related to seasonal variations is possible due to the limited study period. However, a fixed continuous time period was chosen to limit the risk of selection bias. However, the data available from the EHR’s are reliable and conclusive. Finally, we applied the STROBE checklist to our study to reduce the risk of bias.  3-day mortality rate was low, leading to wide confidence intervals, however as it was a priori selected primary outcome, it was included in the results. | | | | | |
| Study size | | 10 | Explain how the study size was arrived at | Convenience sample | | | | | |
| Quantitative variables | | 11 | See Data analysis | |  | | | |  |
| Statistical methods | | 12 | We used the area under receiving operating characteristic (AUROC) analysis for our outcomes: 3-day mortality, 30-day mortality, hospital admission and HDU/ICU admission. The analysis was run for both the standard ESI triage method and a local 3-level HUH method. A further analysis was run for both triage methods with age adjustment: all patient above a certain cut off age were moved into a more urgent triage category. The cut off values used were 65, 70, 75 and 80 years. We calculated the net reclassification improvement (NRI) values to demonstrate the effect of applying age adjustment between triage categories. P-values under 0.05 were considered significant. | | | | | |  |
| Participants | | 13* | Within the study period, there were 15 207 recorded visits to our three ED’s. After excluding patients who were dead on arrival, not seen by a doctor, or who attended for a fracture clinic, we had 13374 who met our study criteria. | | | | | |  |
| Descriptive data | | 14* | See Table 1 | |  | | | |  |
| Outcome data | | 15* | See results | |  | | | |  |
| Main results | | 16 | Confidence intervals were reported. | |  | | | |  |
| Other analyses | 17 | NRI values were reported. | | | |  |  |  |  |
| Key results | 18 | Moving older adults into a more urgent triage category based on age, improved the triage instruments’ performance in predicting 30-day mortality and hospital admission. | | | | | |  |  |
| Limitations | 19 | See strengths and limitations | | | | |  |  |  |
| Interpretation | 20 | See discussion | | | | |  |  |  |
| Generalisability | 21 | See discussion | | | | |  |  |  |
| Other information | |  |  |  |  |  |  |  |  |
| Funding | 22 | See Funding | | | | |  |  |  |

- 1. Appendix 2: Our 3-level triage tool (abbreviated and translated from original Finnish version)

|  | **Red** | **Yellow** | **Green** |
| --- | --- | --- | --- |
| **Dyspnea** | Severe respiratory failure  RR <8 or >30, spO2 <70 | Minimal respiratory failure  RR <25, spO2 >90% | No respiratory failure |
| **Trauma** | Major trauma, major burn | Fractures and dislocations with obvious displacement; | Walking wounded |
| **Bleeding** | Ruptured aortic aneurysm, hematemesis, major gynecological bleeds | Minor/moderate melena, obstetric bleeding | Minor wounds, epistaxis |
| **Chest pain** | Abnormal vital signs, hypovolemic shock, ST-elevation myocardial infarction, chest pain with ST depression | Intermittent chest pain, hemodynamically stable, congestive heart failure | No ECG changes, spontaneously eased chest pain, |
| **Arrhythmias** | Abnormal Glascow coma scale, abnormal vital signs, broad complex tachycardias | Narrow complex tachycardias, arrhythmias with chest pain or dyspnea | Palpitations with normal vital signs and no other symptoms |
| **Altered consciousness and headache** | Unconscious patient, high fever with altered consciousness, status epilepticus, suspected stroke | Acute confusional state, head injury, neck pain, headache | Post convulsion monitoring, vertigo without other symptoms. Transient ischaemic attack. |
| **Abdominal pain** | Shocked patient, major gastrointestinal-bleed, peritonismus, major gynecological bleed, | Bowel obstruction, kidney stone, suspected infection | Jaundice, suspected appendicitis, urinary retention |
| **Back pain** | Suspected spinal cord injury | Back pain with leg weakness or urinary/bowel symptoms or fever | Ambulant; no other symptoms |
| **Eye symptoms** | perforating, thermal, blunt or chemical eye injury, sudden loss of vision | Eye pain, diplopy, temporary loss of vision | Normal vision with eye pain or suspected foreign body |
| **Fever** | Reduced consciousness; abnormal vital signs, shock | Immunocompromised patients; type 1 diabetic; any severe symptoms | Ambulant patients with normal vital signs |
| **Poisoning** | Abnormal vital signs; known beta- or calcium blocker intake | Minor symptoms | - |

# Appendix 3: NRI values for retriaged patients

Interpretation of the NRI table, first row as an example. With the cut off age of 65 for HUH triage, 59% of patients who died were correctly reclassified into higher triage category: NRI (event) was 0.59. 37% of patients who did not die, were incorrectly classified to a higher triage category NRI (non-event) was -0.37. The NRI value is 0.59-0.37 = 0.22.

| **Outcome** | **Triage method** | **Combined** | | | |  | **Event** | | |  | **Non-event** | | |
| --- | --- | --- | --- | --- | --- | --- | --- | --- | --- | --- | --- | --- | --- |
|  |  | **cut off age** | **NRI (95% CI)** | | **p** |  | **NRI (95%CI)** | | **p** |  | **NRI (95%CI)** | | **p** |
| **3-day**  **mortality** | HUH  triage | 65 | 0.22 | (0.02-0.43) | 1.06 |  | 0.59 | (0.39-0.80) | <0.001 |  | -0.37 | (-0.38-0.36) | <0.001 |
|  |  | 70 | 0.24 | (0.03-0.45) | 0.81 |  | 0.55 | (0.34-0.75) | <0.001 |  | -0.31 | (-0.32-0.30) | <0.001 |
|  |  | 75 | 0.33 | (0.12-0.53) | 0.07 |  | 0.55 | (0.34-0.75) | <0.001 |  | -0.22 | (-0.23-0.21) | <0.001 |
|  |  | 80 | 0.17 | (-0.03-0.36) | 3.02 |  | 0.32 | (0.12-0.51) | <0.001 |  | -0.15 | (-0.16-0.14) | <0.001 |
|  | ESI | 65 | 0.13 | (-0.10-0.36) | 9.15 |  | 0.56 | (0.33-0.79) | <0.001 |  | -0.43 | (-0.44-0.42) | <0.001 |
|  |  | 70 | 0.21 | (-0.02-0.44) | 2.36 |  | 0.56 | (0.33-0.79) | <0.001 |  | -0.35 | (-0.36-0.33) | <0.001 |
|  |  | 75 | 0.29 | (0.07-0.52) | 0.38 |  | 0.56 | (0.33-0.79) | <0.001 |  | -0.26 | (-0.27-0.25) | <0.001 |
|  |  | 80 | 0.37 | (0.14-0.60) | 0.05 |  | 0.56 | (0.33-0.79) | <0.001 |  | -0.19 | (-0.10-0.18) | <0.001 |
| **30-day**  **mortality** | HUH  triage | 65 | 0.32 | (0.24-0.39) | <0.001 |  | 0.68 | (0.61-0.75) | <0.001 |  | -0.36 | (-0.37-0.35) | <0.001 |
|  |  | 70 | 0.30 | (0.23-0.38) | <0.001 |  | 0.61 | (0.53-0.68) | <0.001 |  | -0.30 | (-0.31-0.39) | <0.001 |
|  |  | 75 | 0.32 | (0.24-0.40) | <0.001 |  | 0.53 | (0.45-0.61) | <0.001 |  | -0.21 | (-0.22-0.20) | <0.001 |
|  |  | 80 | 0.25 | (0.17-0.33) | <0.001 |  | 0.40 | (0.32-0.48) | <0.001 |  | -0.15 | (-0.16-0.14) | <0.001 |
|  | ESI | 65 | 0.32 | (0.25-0.40) | <0.001 |  | 0.75 | (0.68-0.82) | <0.001 |  | -0.42 | (-0.44-0.41) | <0.001 |
|  |  | 70 | 0.32 | (0.25-0.40) | <0.001 |  | 0.66 | (0.58-0.74) | <0.001 |  | -0.34 | (-0.35-0.33) | <0.001 |
|  |  | 75 | 0.33 | (0.25-0.41) | <0.001 |  | 0.58 | (0.50-0.66) | <0.001 |  | -0.25 | (-0.26-0.24) | <0.001 |
|  |  | 80 | 0.31 | (0.23-0.39) | <0.001 |  | 0.49 | (0.41-0.57) | <0.001 |  | -0.18 | (-0.19-0.17) | <0.001 |
| **Hospital**  **admission** | HUH  triage | 65 | 0.27 | (0.25-0.29) | <0.001 |  | 0.56 | (0.54-0.58) | <0.001 |  | -0.29 | (-0.20-0.28) | <0.001 |
|  |  | 70 | 0.26 | (0.24-0.28) | <0.001 |  | 0.49 | (0.47-0.51) | <0.001 |  | -0.23 | (-0.24-0.22) | <0.001 |
|  |  | 75 | 0.22 | (0.20-0.25) | <0.001 |  | 0.38 | (0.36-0.40) | <0.001 |  | -0.16 | (-0.16-0.15) | <0.001 |
|  |  | 80 | 0.18 | (0.16-0.20) | <0.001 |  | 0.28 | (0.26-0.30) | <0.001 |  | -0.10 | (-0.11-0.19) | <0.001 |
|  | ESI | 65 | 0.32 | (0.29-0.34) | <0.001 |  | 0.62 | (0.60-0.64) | <0.001 |  | -0.30 | (-0.32-0.38) | <0.001 |
|  |  | 70 | 0.28 | (0.26-0.31) | <0.001 |  | 0.51 | (0.49-0.53) | <0.001 |  | -0.23 | (-0.25-0.22) | <0.001 |
|  |  | 75 | 0.24 | (0.22-0.27) | <0.001 |  | 0.41 | (0.39-0.43) | <0.001 |  | -0.16 | (-0.17-0.15) | <0.001 |
|  |  | 80 | 0.20 | (0.18-0.23) | <0.001 |  | 0.31 | (0.29-0.33) | <0.001 |  | -0.11 | (-0.12-0.19) | <0.001 |
| **HDU/ICU**  **admission** | HUH  triage | 65 | 0.11 | (0.07-0.16) | <0.001 |  | 0.47 | (0.43-0.52) | <0.001 |  | -0.36 | (-0.37-0.35) | <0.001 |
|  |  | 70 | 0.10 | (0.05-0.14) | 0.001 |  | 0.40 | (0.36-0.44) | <0.001 |  | -0.30 | (-0.31-0.39) | <0.001 |
|  |  | 75 | 0.07 | (0.03-0.11) | 0.032 |  | 0.28 | (0.24-0.32) | <0.001 |  | -0.22 | (-0.22-0.21) | <0.001 |
|  |  | 80 | 0.06 | (0.02-0.10) | 0.050 |  | 0.21 | (0.17-0.24) | <0.001 |  | -0.15 | (-0.16-0.14) | <0.001 |
|  | ESI | 65 | 0.09 | (0.02-0.17) | 0.345 |  | 0.52 | (0.45-0.59) | <0.001 |  | -0.43 | (-0.44-0.41) | <0.001 |
|  |  | 70 | 0.03 | (-0.04-0.10) | 10.93 |  | 0.38 | (0.31-0.45) | <0.001 |  | -0.34 | (-0.36-0.33) | <0.001 |
|  |  | 75 | 0.01 | (-0.06-0.07) | 26.42 |  | 0.27 | (0.21-0.33) | <0.001 |  | -0.26 | (-0.27-0.25) | <0.001 |
|  |  | 80 | -0.01 | (-0.07.0.05) | 23.37 |  | 0.18 | (0.12-0.23) | <0.001 |  | -0.19 | (-0.10-0.18) | <0.001 |
